# Targeting α7 nicotinic acetylcholine receptors for chronic pain

**DOI:** 10.3389/fnmol.2022.970040

**Published:** 2022-09-30

**Authors:** Ya-Qun Zhou, Dai-Qiang Liu, Cheng Liu, Ai-Jun Xu, Yu-Ke Tian, Wei Mei, Xue-Bi Tian

**Affiliations:** Department of Anesthesiology and Pain Medicine, Tongji Hospital, Tongji Medical College, Huazhong University of Science and Technology, Wuhan, China

**Keywords:** chronic pain, α7 nAChR, neuroinflammation, microglia, oxidative stress

## Abstract

Despite rapid advances in the field of chronic pain, it remains extremely challenging in the clinic. Pain treatment strategies have not improved for decades as opioids remain the main prescribed drugs for chronic pain management. However, long-term use of opioids often leads to detrimental side effects. Therefore, uncovering the mechanisms underlying the development and maintenance of chronic pain may aid the discovery of novel therapeutics to benefit patients with chronic pain. Substantial evidence indicates downregulation of α7 nicotinic acetylcholine receptors (α7 nAChR) in the sciatic nerve, dorsal root ganglia, and spinal cord dorsal horn in rodent models of chronic pain. Moreover, our recent study and results from other laboratories demonstrate that potentiation of α7 nAChR attenuates pain behaviors in various murine models of chronic pain. This review summarized and discussed the preclinical evidence demonstrating the therapeutic potential of α7 nAChR agonists and allosteric modulators in chronic pain. This evidence indicates that potentiation of α7 nAChR is beneficial in chronic pain, mostly by alleviating neuroinflammation. Overall, α7 nAChR-based therapy for chronic pain is an area with great promise, but more research regarding its detailed mechanisms is warranted.

## Introduction

Despite rapid advances in the field of chronic pain, it remains extremely challenging in the clinic (Burma et al., 2017; Cornett et al., 2018). Pain treatment strategies have not improved for decades as opioids remain the main prescribed drugs for chronic pain management (Matthias et al., 2020; Goldstick et al., 2022). However, long-term use of opioids often leads to detrimental side effects, including respiratory depression, mental clouding, tolerance, addiction, constipation, nausea, and vomiting (Liu et al., 2019). Therefore, uncovering the mechanisms underlying the development and maintenance of chronic pain may aid the discovery of novel therapeutics to benefit patients with chronic pain.

Nicotinic acetylcholine receptors (nAChRs), a member of the cysteine-loop (Cys-loop) family of ligand-gated ion channels, are involved in various physiological and pathological processes (Matta et al., 2021). Notably, the nAChRs are distributed on the pain transmission pathways (Hone and Mcintosh, 2018). Emerging evidence indicates that α4β2, α7, and α9α10 nAChR subtypes are promising therapeutic targets for the management of chronic pain (Dineley et al., 2015; Papke and Lindstrom, 2020). In particular, α7 nAChR is one of the most abundant nAChR in the central nervous system and the peripheral system, as well as immune cells (Papke and Horenstein, 2021). Growing shreds of evidence indicate that potentiation of α7 nAChR is a novel therapeutic strategy for neurological diseases, including Alzheimer's diseases and Parkinson's diseases, schizophrenia, and traumatic brain injury (Kelso and Oestreich, 2012; Beinat et al., 2015; Quik et al., 2015; Ma and Qian, 2019). It has been suggested that activation of α7 nAChR exhibits anti-inflammatory properties (Egea et al., 2015; Zhu et al., 2021). Additionally, it is well-established that α7 nAChR plays a vital role in modulating neuroinflammation, which is one of the main characterizations of chronic pain (Echeverria et al., 2016; Mizrachi et al., 2021). Neuroinflammation in chronic pain is characterized by activation of glial cells, overproduction of proinflammatory cytokines, and infiltration of immune cells in the peripheral and central nervous system (Ji et al., 2014, 2018; Liu et al., 2019). It was reported that α7 nAChR potentiation significantly inhibited the activation of microglia and astrocytes in the spinal cord of rodent models of chronic pain (Sun et al., 2019; Micheli et al., 2021). Moreover, α7 nAChR agonists and allosteric modulators suppressed the release of proinflammatory cytokines in chronic pain models (Loram et al., 2010; Sun et al., 2017). More importantly, α7 nAChR agonists and allosteric modulators have shown potent analgesic effects (Bagdas et al., 2018b; Quadri et al., 2018; Wang et al., 2019; Arias et al., 2020; Han et al., 2020). Therefore, it is plausible that potentiation of the α7 nAChR may be a promising therapeutic strategy for the management of chronic pain. Notably, it is worth mentioning that Bagdas et al. (2018a) provided the readers with insights on α7 nAChRs from structure and function to findings on the pharmacology and therapeutic targeting of α7 nAChRs for the treatment of pain and inflammation in 2018. In this review, we discussed the therapeutic potential of α7 nAChR agonists and allosteric modulators in chronic pain in preclinical studies while identifying numerous crucial problems that need to be addressed.

## The role of α7 nAChR in chronic pain

It has been reported that α7 nAChR is expressed on neuronal cells in the dorsal root ganglia (DRG), spinal cord, and brain, as well as non-neuronal cells in the pain transmission pathway (Cordero-Erausquin et al., 2004). The protein level of α7 nAchR was significantly downregulated in the sciatic nerve, DRG, and spinal cord in oxaliplatin-induced neuropathic pain rats (Di Cesare Mannelli et al., 2014). Similarly, the protein level of α7 nAchR was considerably decreased after single prolonged stress (SPS) exposure in the spinal cord (Sun et al., 2019). Our recent study also revealed that the expression of spinal α7 nAChR was significantly downregulated in cancer-induced bone pain (CIBP) rats, and most of the α7 nAChR was localized in neurons (Yang et al., 2021). The ventrolateral periaqueductal gray (vlPAG) and rostral ventromedial medulla (RVM) are the major components of the descending pain modulatory pathway, which are important targets of analgesic drugs (Bagley and Ingram, 2020). Umana et al. (2017) showed that 63% of PAG-RVM projection neurons expressed functional nAChR, which were exclusively the α7-subtype. These results indicate that decreased expression of α7 nAChR in the pain transmission pathway might be involved in the development of chronic pain.

Compelling evidence from our laboratory and others has demonstrated that activation of α7 nAchR could attenuate chronic pain, including neuropathic pain, inflammatory pain, and CIBP (Medhurst et al., 2008; Papke et al., 2015; Arias et al., 2020; Yang et al., 2021). Alsharari et al. evaluated pain behaviors in the α7 mutant mice (KO) and α7 hypersensitive mice (KI) expressing the L250T α7 nAChR and their respective WT mice in chronic pain models (Alsharari et al., 2013). While no significant change was observed between α7 KO mice and WT mice regarding thermal and mechanical allodynia after chronic nerve injury, α7 KI mice showed a significant reduction in these pain behaviors. Moreover, α7 KO mice displayed a marked increase in edema, hyperalgesia, and allodynia after intraplantar injection of complete Freund adjuvant (CFA). Importantly, systemic administration of nicotine reversed established mechanical allodynia after CFA in WT mice, which was lost in the α7 KO mice. These results indicate that endogenous α7 nAChR plays a vital role in chronic pain. Recently, Khasabov et al. (2021) investigated whether loss of neuronal-specific TMEM35a, a novel chaperone for functional expression of the homomeric α7, in the spinal cord modulates pain in mice. They found that mice with tmem35a deletion exhibited thermal hyperalgesia and mechanical allodynia. Furthermore, they found that the spinal cord of tmem35a KO mice exhibited 72 differentially expressed genes compared with WT control, which was associated with neuroinflammation. Overall, these results indicate a promising therapeutic potential of α7 nAchR in chronic pain.

## The therapeutic potential of α7 nAChR agonists and allosteric modulators in chronic pain

Nicotine modifies the activity of nAChRs, including α7 nAchR. α7 nAchR can also be activated by selective agonists (e.g., GTS-21 and TC-7020) and allosteric modulators (e.g., PNU-120596 and GAT107). The remainder of this review will provide detailed insight into the therapeutic potential of α7 nAChR agonists and allosteric modulators in chronic pain.

### Nicotine

It has been frequently reported that nicotine displays potent antinociceptive effects both in human and rodent studies (Richardson et al., 2012; Di Cesare Mannelli et al., 2013; Brunori et al., 2018). Costa et al. (2012) showed that both acute and chronic oral treatments with nicotine remarkably inhibited established mechanical allodynia in dextran sulfate sodium (DSS)-induced visceral pain in mice. Moreover, the antinociceptive effect of nicotine was completely abolished by co-treatment with methyllycaconitine (MLA), a selective α7 nAchR antagonist. Nevertheless, nicotine did not affect DSS-induced colonic damage and inflammation. In another study, Teng et al. (2019) demonstrated that intraperitoneal injection of nicotine considerably attenuated mechanical allodynia, cartilage degradation, and upregulation of matrix metalloproteinase-9 in monosodium iodoacetate (MIA)-induced osteoarthritis pain mice, which were entirely abolished by MLA. Their further study validated that nicotine alleviated MIA-induced pain behaviors and cartilage degradation *via* stimulating the α7 nAChR/mammalian target of the rapamycin signal pathway (Liu et al., 2021). These results indicate that nicotine attenuates chronic pain in an α7 nAchR-dependent manner. Nevertheless, it is worth noting that long-term nicotine administration led to mechanical allodynia, which could be reversed by the selective α7 nAChR agonist CDP-Choline (Zhang et al., 2021c).

### Selective α7 nAChR agonists

The role of α7 nAChR in chronic pain was not fully elucidated until the discovery of selective agonists. Medhurst et al. revealed that intraperitoneal injection of (R)-N-(1-azabicyclo[2.2.2]oct-3-yl) (5-(2-pyridyl) thiophene-2-carboxamide) (compound B), a selective α7 nAChR agonist, completely reversed CFA-induced inflammatory pain in mice and rats in a dose-related manner (Medhurst et al., 2008). Importantly, subcutaneous treatment with selective low-brain penetrant α7 antagonist MLA did not affect compound B activity, while intrathecal injection of MLA completely inhibited the agonist effect. These results indicate that compound B alleviated inflammatory pain *via* activation of α7 nAChR in the central nervous system. In another study, Loram et al. (2010) showed that intrathecal injection of choline, a precursor for acetylcholine and a selective α7 nAChR agonist, considerably suppressed and reversed gp120-induced mechanical allodynia and upregulation of proinflammatory cytokines, including TNFα, IL-1β, and IL6 in the spinal cord. Moreover, they found that GTS-21, another selective α7 nAchR agonist, exhibited similar properties. Furthermore, both choline and GTS-21 treatment inhibited the activation of microglia in the spinal cord, as indicated by decreased expression of cd11b. These results indicate that α7 nAchR agonists might attenuate neuropathic pain *via* suppressing neuroinflammation. Consistently, Zhang et al. (2021b) demonstrated that microinjection of α7 nAChR siRNA into ipsilateral L4/5 DRG aggravated CFA-induced inflammatory pain, while intrathecal injection of GTS-21 attenuated the development of CFA-induced inflammatory pain. Their *in vitro* study showed that knockdown of α7 nAChR initiated the activation of TNF receptor-associated factor 6 (TRAF6) and nuclear factor kappa B (NF-κB) under CFA-induced inflammatory conditions, while α7 nAChR activation inhibited the upregulation of TRAF6 and NF-κB. These results indicate that GTS-21 might attenuate inflammatory pain by inhibiting the TRAF6/NF-κB signaling pathway. In a sciatic chronic constriction injury (CCI)-induced neuropathic pain model, Loram et al. (2012) demonstrated that subcutaneous injection of TC-7020, a selective α7 nAChR agonist, significantly attenuated CCI-induced mechanical allodynia. Moreover, TC-7020 downregulated the integrated density of activation transcription factor 3 and phosphorylated extracellular signal kinase as well as satellite cell activation in DRG in CCI rats. These results indicate that systemic administration of TC-7020 may attenuate neuropathic pain by reducing neuronal injury and immune cell activation in the DRG.

In a mouse model of visceral pain, Costa et al. (2012) showed that intraperitoneal injection of selective α7 nAchR agonist PNU-282987 significantly reduced DSS-induced mechanical allodynia. Similarly, Di Cesare Mannelli et al. (2014) demonstrated that PNU-282987 significantly reduced mechanical allodynia in oxaliplatin-treated rats. Importantly, PNU-282987 treatment prevented the downregulation of α7 nAchR in the sciatic nerve, DRG, and spinal cord in oxaliplatin-induced neuropathic pain rats. In our recent study, we further investigated the analgesic effect of PNU-282987 in a rat model of CIBP (Yang et al., 2021). We found that both acute and chronic treatment with PNU-282987 significantly attenuated mechanical allodynia in CIBP rats, which was completely blocked by pretreatment with MLA. Moreover, repeated administration of PNU-282987 reversed the downregulation of α7 nAChR and inhibited the upregulation of NF-κB in the spinal cord. These findings indicate that PNU-282987 might attenuate chronic pain by restoring the expression of α7 nAChR. In another study, Sun et al. (2017) demonstrated that intrathecal injection of PHA-543613, a selective α7 nAChR agonist, dose-dependently attenuated SPS-evoked mechanical allodynia, which was remarkably blocked by pretreatment with MLA.

Moreover, SPS-induced glial activation and upregulation of proinflammatory cytokines were considerably suppressed by PHA-543613, which was abolished by MLA. Their further study confirmed that repeated intrathecal injection of PHA-543613 during the perioperative period shortened the duration of post-surgical pain after SPS and suppressed SPS-potentiated microglia activation, which was also abolished by pretreatment with MLA (Sun et al., 2019). Consistently, Ji et al. (2019) validated that intrathecal injection of PHA-543613 significantly alleviated spinal nerve ligation-induced neuropathic pain by decreasing dynorphin A concentration in the ipsilateral spinal cord. Furthermore, Umana et al. (2017) revealed that both systemic and intra-vlPAG administration of PHA-543613 significantly attenuated formalin-induced inflammatory pain, which was completely abolished by pretreatment with MLA. These findings indicate that PHA-543613 is a promising therapeutic strategy for the management of chronic pain. Recently, Micheli et al. (2021) investigated the protective effects of DDD-028, a versatile pentacyclic pyridoindole derivative, against paclitaxel-induced neuropathic pain. Both acute and chronic treatment with DDD-028 dose-dependently attenuated mechanical allodynia in paclitaxel-treated rats, which was entirely blocked by MLA.

Moreover, DDD-028 alleviated oxidative stress in DRG, as evidenced by the increased level of carbonylated proteins and decreased catalase activity. Importantly, DDD-28 significantly prevented the activation of microglia and astrocytes in the lumbar spinal cord, periaqueductal gray matter, thalamus, and somatosensory cortex. These findings suggest that DDD-028 might be a valuable candidate for the treatment of chemotherapy-induced peripheral neuropathy. Collectively, these results provide solid evidence demonstrating the analgesic effects of selective α7 nAChR agonists.

### α7 nAChR positive allosteric modulators

Despite the encouraging therapeutic efficacy of α7 nAChR agonists in chronic pain, concerns have been raised regarding the long-term administration of α7 nAChR agonists due to rapid desensitization of α7 nAChR, receptor selectivity issues, and the narrow window of antinociceptive effect *in vivo* (Freitas et al., 2013a; Toma et al., 2020). These limitations hinder the application of α7 nAChR as analgesics in the clinic. However, they led to the development of α7 nAChR positive allosteric modulators (PAMs), which potentiate α7 currents in the presence of an endogenous agonist such as acetylcholine and choline. Type I PAMs facilitate agonist response with little effect on desensitization of α7 nAChRs, while type II PAMs facilitate agonist response and retard the obvious desensitization profile of the agonist response (Toma et al., 2020). Freitas et al. (2013b) investigated two types of PAMs in chronic pain models. They found that both NS1738 (type I) and PNU-120596 (type II) considerably attenuated thermal hyperalgesia, while only PNU-120596 considerably reduced edema in carrageenan-induced inflammatory pain mice. Moreover, PNU-120596 reversed established thermal hyperalgesia and edema induced by carrageenan. Additionally, a single dose of PNU-120596 significantly attenuated mechanical allodynia and thermal hyperalgesia in CCI mice, while NS1738 was ineffective. Importantly, the analgesic effect of PNU-120596 was completely blocked by systemic administration of the α7 nAChR antagonist MLA. Furthermore, an ineffective dose of selective α7 nAChR agonist PHA-543613 produced anti-allodynic effects in CCI mice by co-administration of PNU-120596. Their further study demonstrated that systemic administration of PNU-120596 dose-dependently attenuated nociceptive behaviors in the formalin test (Freitas et al., 2013c). Moreover, PNU-120596 enhanced the effects of nicotine and PHA-543613 in the formalin test. Our recent study also demonstrated that PNU-120596 enhanced the analgesic effect of PNU-282987 in a rat model of CIBP (Yang et al., 2021). Consistently, Sun et al. demonstrated that repeated intrathecal injection of PNU-120596 during the perioperative period shortened the duration of post-surgical pain after SPS and suppressed SPS-potentiated microglia activation, which was markedly abolished by pretreatment with MLA (Sun et al., 2019). These results indicate that type II α7 nAChR PAM PNU-120596 shows a potent analgesic effect in chronic pain. In another study, Bagdas et al. (2016) investigated the antinociceptive role of GAT107, a potent α7 nAChR type II PAM with intrinsic allosteric agonist activities. They found that GAT107 dose-dependently attenuated CFA-induced inflammatory pain and CCI-induced neuropathic pain. Moreover, intrathecal, but not intraplantar, injection of GAT107 reversed established pain behaviors in CFA mice. Furthermore, intrathecal GAT107 treatment inhibited the activation of astrocytes and upregulated p38 mitogen-activated protein kinase in the spinal cord. In a mouse model of lipopolysaccharide (LPS)-induced inflammatory pain, Abbas et al. (2019) investigated the analgesic effect of 3a,4,5,9b-tetrahydro-4-(1-naphthalenyl)-3H-cyclopentan[c]quinoline-8-sulfonamide (TQS), an α7 nAChR type II PAM. They found that systemic administration of TQS prevented LPS-induced mechanical allodynia and thermal hyperalgesia by decreasing the expression of Iba-1, p-NF-κB, and TNF-α in CA1 and dentate gyrus regions of the hippocampus. Their further study demonstrated that TQS attenuated LPS-induced inflammatory pain *via* downregulation of hippocampal brain-derived neurotrophic factor and Na^+^.K^+^.2Cl^−^ and upregulation of K^+^.Cl^−^ (Abbas et al., 2021). Recently, Bagdas et al. (2021) demonstrated that acute systemic administration of PAM-4, a highly selective α7-nAChR PAM, dose-dependently reversed formalin-induced inflammatory pain and CCI-induced neuropathic pain without the development of any motor impairment, which was entirely blocked by MLA. Moreover, PAM-4 reversed CCI-induced depression-like behavior and anxiogenic-like effects. These results indicate that PAM-4 reduces both sensorial and affective behaviors in chronic pain *via* α7 nAChR potentiation. Interestingly, secreted mammalian Ly6/urokinase plasminogen activator receptor-related protein-1 (SLURP-1) has been identified as an endogenous ligand of α7 nAChR (Chimienti et al., 2003). It was reported that SLURP-1 is expressed in the dorsal horn of the spinal cord and a subset of primary peptidergic sensory neurons in the dorsal root ganglia (Moriwaki et al., 2009). Therefore, it is plausible that SLURP-1 might play a role in chronic pain *via* activation of α7 nAChR. Collectively, these results indicate a promising future for the management of chronic pain *via* α7 nAChR positive allosteric modulators.

### α7 nAChR silent agonists

Silent agonists are an additional class of α7 nAChR ligands (Papke et al., 2014). Toma et al. (2019) demonstrated that R-47, an α7 nAChR silent agonist, prevented and reversed paclitaxel-induced mechanical allodynia in mice in an α7 nAChR-dependent manner. Moreover, R-47 prevented paclitaxel-induced loss of intraepidermal nerve fibers and activation of microglia in the spinal cord. Importantly, R-47 did not affect tumor cell viability or interfere with the antitumor activity of paclitaxel in tumor-bearing mice. These results indicate that R-47 might be a promising strategy for preventing and treating paclitaxel-induced neuropathic pain.

## Concluding remarks and future perspective

**Figure 1:**
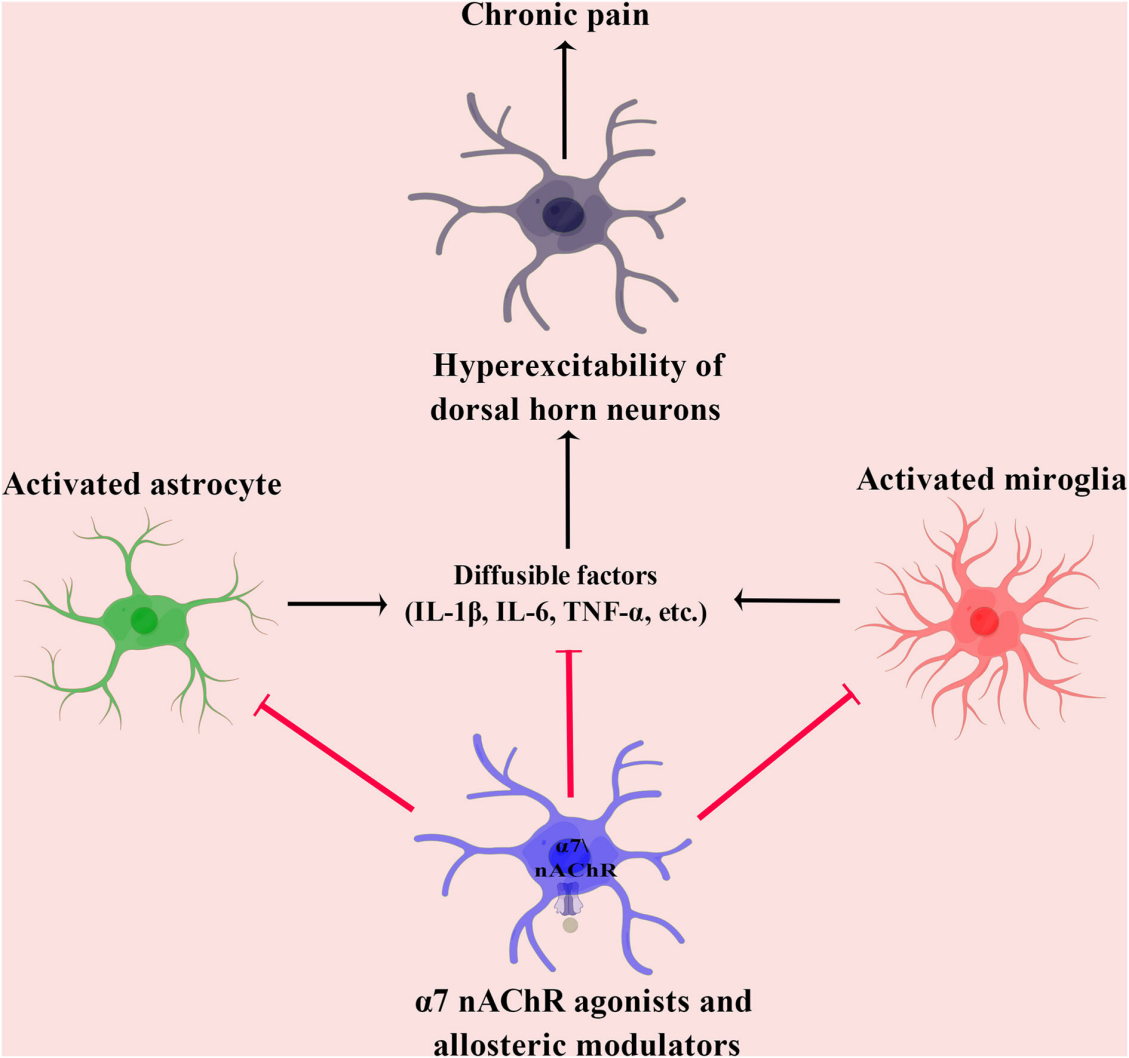
In preclinical studies, the mechanisms underlying the analgesic effect of α7 nAChR agonists and allosteric modulators in chronic pain. IL-1β, interleukin 1β; IL-6, interleukin 6; nAChR, nicotinic acetylcholine receptor; and TNF-α, tumor necrosis factor-α.

Data from our laboratory and others have shown decreased expression of α7 nAChR in the pain transmission pathway. Notably, the potentiation of α7 nAChR exerts a potent analgesic effect against chronic pain in preclinical studies (Gong et al., 2015; Criado et al., 2016; Quadri et al., 2018; Yang et al., 2018; Apryani et al., 2020). This review summarized and discussed the therapeutic potential of α7 nAChR agonists and allosteric modulators in chronic pain (Table 1). This evidence showed that potentiation of α7 nAChR attenuates chronic pain mainly through modulating neuroinflammation (Figure 1). However, these findings raise further questions.

**Table 1:** Summary of the therapeutic potential of α7 nAChR agonists and allosteric modulators in chronic pain.

**Compound**	**Chemical structure**	**Model**	**Treatment strategy**	**Effects**	**Mechanisms**	**References**
Nicotine	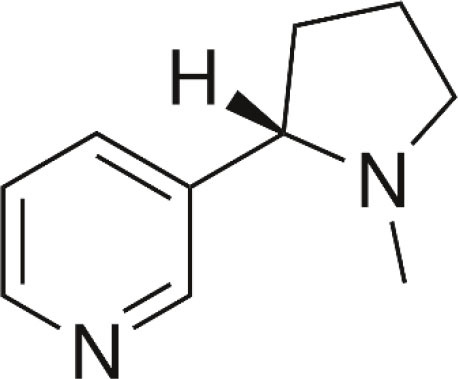	MIA-induced osteoarthritis pain mice	Nicotine (0.5 and 1.0 mg/kg, i.p.) was administered once daily for seven consecutive days before MIA and 21 straight days after MIA.	PWT↑	Activation of α7 nAChR	Teng et al., 2019
DSS-induced visceral pain mice		Nicotine (0.1, 0.3, and 1.0 mg/kg, p.o.) was administered two times daily at 12-h intervals from day 3 to 6 of 4% DSS intake.	PNT↑ ANT↑	Activation of α7 nAChR	Costa et al., 2012
Choline	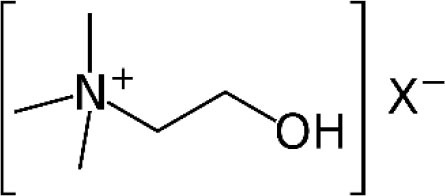	gp120-induced neuropathic pain rats	Choline (0.1, and 1 μM, i.t.) was administered intrathecally either with or 30 min after intrathecal gp120.	PWT↑	Activation of α7 nAChR Microglial activation↓ TNFα, IL-1β and IL6 ↓	Loram et al., 2010
Compound B	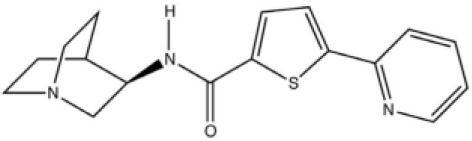	CFA-induced inflammatory pain in rats and mice	Compound B (1, 3, and 10 mg/kg, i.p.) was administered 23.5 h after CFA injection in rats. Compound B (5, 10, and 20 mg/kg, i.p.) was administered 23.5 h after CFA injection in mice.	PWT↑ DWB↓	Activation of α7 nAChR	Medhurst et al., 2008
GTS-21	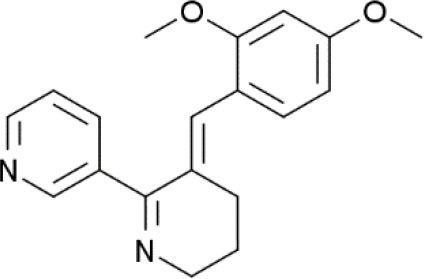	CFA-induced inflammatory pain mice	GTS-21 (5,000, 10,000, and 20,000 nM/mice, i.t.) was injected on day 3 after the CFA injection.	PWL↑	Activation of α7 nAChR TRAF6, NF-κB↓	Zhang et al., 2021b
gp120-induced neuropathic pain rats		GTS-21 (1, and 10 μM, i.t.) was administered intrathecally either with or 30 min after intrathecal gp120.	PWT↑	Activation of α7 nAChR Microglial activation↓ TNFα, IL-1β and IL6 ↓	Loram et al., 2010
TC-7020	/	CCI-induced neuropathic pain rats	TC-7020 (1, 3, and 10 mg/kg/d, s.c.) was administered *via* osmotic mini-pumps for 14 days starting from day 10 after surgery.	PWT↑	Activation of α7 nAChR Neuronal injury ↓ Immune cells activation ↓	Loram et al., 2012
PNU-282987	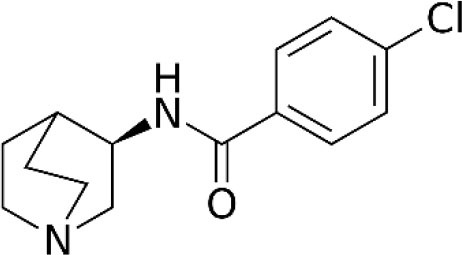	Oxaliplatin-induced neuropathic pain rats	PNU-282987 (30 mg/kg, p.o.) was administered acutely on day 21 or daily starting from the first day of oxaliplatin administration up to day 20.	PWT↑ PWL↑	Activation of α7 nAChR	Di Cesare Mannelli et al., 2014
		Cancer-induced bone pain rats	PNU-282987 (0.1, 0.25, and 0.5 mg/kg, i.t.) was given on day 14 after surgery. PNU-282987 (0.5 mg/kg, i.t.) was given once daily from day 14 to 18 after surgery.	PWT↑	Activation of α7 nAChR NF-κB↓	Yang et al., 2021
		DSS-induced visceral pain mice	PNU-282987 (0.1, 0.3, and 1.0 mg/kg, i.p.) was administered two times a day at 12-h intervals from day 3 to 6 of 4% DSS intake.	PNT↑	Activation of α7 nAChR	Costa et al., 2012
PHA-543613	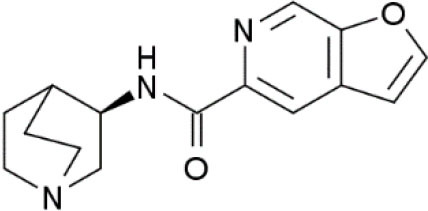	Formalin-induced inflammatory pain rats	PHA-543613 (0.3 and 3 nmol, Intra-vlPAG) was administered 5 min before formalin injection. PHA-543613 (0.2–10 mg/kg, s.c.) was administered 15 min before formalin injection.	PWL↑	Activation of α7 nAChR	Umana et al., 2017
		SPS-induced chronic pain rats	PHA-543613 (6 and 12 μg, i.t.) was administered for 8 consecutive days starting from the day of SPS. PHA-543613 (12 μg, i.t.) was administered on day 7 after SPS.	PWT↑	Activation of α7 nAChR Microglial activation↓ Astrocytic activation↓ TNFα and IL-1β↓	Sun et al., 2017
		SNL-induced neuropathic pain rats	PHA-543613 (12 μg, i.t.) was administered 7 or 21 days after SNL.	PWT↑ PWL↑	Activation of α7 nAChR TNFα and IL-1β↓ dynorphin A↓	Ji et al., 2019
		Preoperative stress-induced prolongation of postsurgical pain rats	PHA-543613 (12 μg, i.t.) was administered 30 min before SPS or on the first day after incisional surgery. PHA-543613 (12 μg, i.t.) was administered once daily for 5 consecutive days starting from 30 min before SPS.	PWT↑	Activation of α7 nAChR Microglial activation↓ TNFα and IL-1β↓ NF-κB↓	Sun et al., 2019
DDD-028	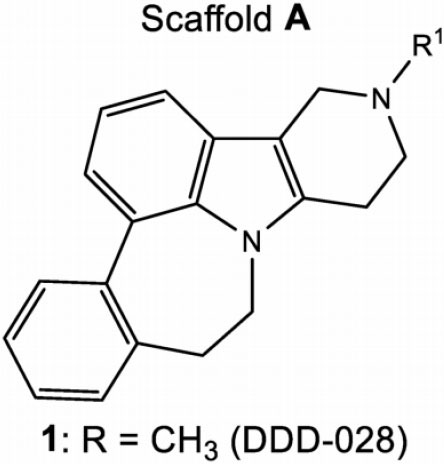	Paclitaxel-induced neuropathic pain rats	DDD-028 (1, 5, 10, and 25 mg/kg, p.o.) was administered on day 10 after the initial injection of paclitaxel. DDD-028 (10 mg/kg, p.o.) was administered twice daily for 18 consecutive days starting from the beginning of the paclitaxel injection.	PWT↑ PWL↑	Activation of α7 nAChR Oxidative stress↓ Microglial activation↓ Astrocytic activation↓	Micheli et al., 2021
PNU-120596	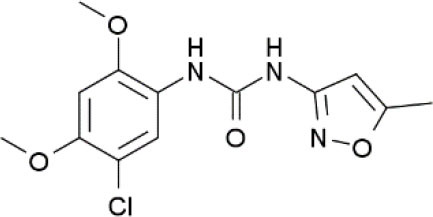	Carrageenan-induced inflammatory pain mice	PNU-120596 (1 and 4 mg/kg, s.c.) was administered 15 min before intraplantar injection of carrageenan. PNU-120596 (8 mg/kg, i.p.) was administered 3 h after carrageenan.	PWL↑	Activation of α7 nAChR	Freitas et al., 2013b
		CCI-induced neuropathic pain mice	PNU-120596 (1, 2, and 4 mg/kg, i.p.) was administered 10 days after CCI.	PWT↑ PWL↑	Activation of α7 nAChR	Freitas et al., 2013b
		Preoperative stress-induced prolongation of postsurgical pain rats	PNU-120596 (15 μg, i.t.) was administered once daily for 5 consecutive days starting from 30 min before SPS.	PWT↑	Activation of α7 nAChR Microglial activation↓ TNFα and IL-1β↓ NF-κB↓	Sun et al., 2019
GAT107	/	Formalin-induced inflammatory pain mice	GAT107 (0.1, 1, 3, and 10 mg/kg, i.p.) was injected 15 min before formalin injection. Mice were administered with GAT107 (1 and 10 mg/kg, i.p.) for 6 days twice daily with 8 h apart and were challenged with GAT107 (1 or 10 mg/kg, i.p.) on day 7 and tested in the formalin test.	PWL↑	Activation of α7 nAChR	Bagdas et al., 2016
		CFA-induced inflammatory pain mice	GAT107 (1, 3, and 10 mg/kg, i.p.) was injected on day 3 after the CFA injection. GAT107 (0.3 and 3 μg/5 μL/mouse, i.t.) was injected on day 3 after CFA injection. GAT107 (3 and 9 μg/20 μL/mouse, i.pl.) was injected on day 3 after CFA injection.	PWT↑ PWL↑	Activation of α7 nAChR Astrocytic activation↓ p38 MAPK↓	Bagdas et al., 2016
		CCI-induced neuropathic pain mice	GAT107 (1, 3, and 10 mg/kg, i.p.) was injected 2 weeks after CCI surgery.	PWT↑ PWL↑	Activation of α7 nAChR	Bagdas et al., 2016
TQS	/	LPS-induced inflammatory pain	TQS (0.25, 1, and 4 mg/kg, i.p.) was given 30 min before LPS administration.	PWT↑ PWL↑	Activation of α7 nAChR Microglial activation↓ NF-κB, TNFα↓	Abbas et al., 2019
		LPS-induced inflammatory pain	TQS (1 and 4 mg/kg, i.p.) was given 30 min before LPS administration.	PWT↑ PWL↑	Activation of α7 nAChR BDNF, NKCC1↓ KCC2↑	Abbas et al., 2021
PAM-4	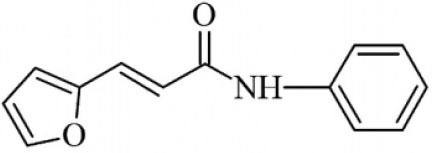	Formalin-induced inflammatory pain mice	PAM-4 (1, 2, and 4 mg/kg, i.p.) was administered 15 min before the formalin injection.	TSL↓	Activation of α7 nAChR	Bagdas et al., 2021
		CCI-induced neuropathic pain mice	PAM-4 (1 and 2 mg/kg, i.p.) was administered 2–3 weeks after CCI surgery.	PWT↑	Activation of α7 nAChR	Bagdas et al., 2021
R-47	/	Paclitaxel-induced neuropathic pain mice	R-47 (1, 5, and 10 mg/kg, p.o.) was administered on day 7 after the initial injection of paclitaxel. R-47 (10 mg/kg, p.o.) was administered twice daily for 3 consecutive days before and during the paclitaxel injection cycle.	PWT↑	Activation of α7 nAChR Microglial activation↓ IENFs↑	Toma et al., 2019

ANT, abdominal nociceptive threshold; BDNF, brain-derived neurotrophic factor; CCI, chronic constriction injury; CFA, complete Freund's adjuvant; DSS, dextran sulfate sodium; DWB, difference in weight bearing between both hind limbs; i.g., intragastrically; i.p., intraperitoneally; i.pl., intraplantarly; i.t., intrathecally; IL-1β, interleukin-1β; IL-6, interleukin 6; KCC2, K^+^.Cl^−^; LPS, lipopolysaccharide; MAPK, mitogen-activated protein kinase; MIA, monosodium iodoacetate; nAChR, nicotinic acetylcholine receptor; NF-κB, nuclear factor kappa B; NKCC1, Na^+^.K^+^.2Cl^−^; p.o., per oral; PNT, paw nociceptive threshold; PWT, paw withdrawal threshold; s.c., subcutaneously; SNL, spinal nerve ligation; SPS, single prolonged stress; TNFα, tumor necrosis factor α; TQS, 3a,4,5,9b-tetrahydro-4-(1-naphthalenyl)-3H-cyclopentan[c]quinoline-8-sulfonamide; TRAF6, tumor necrosis factor receptor-associated factor 6; TSL, time spent in licking; vlPAG, ventrolateral periaqueductal gray; ↓, downregulated; ↑, upregulated.

First of all, most of the studies mentioned above focused on behavioral tests after treatment rather than the underlying mechanisms. It is well-established that neuroinflammation plays a pivotal role in the development of chronic pain (Zhou et al., 2019; Jiang et al., 2020; Chen et al., 2021). The studies mentioned above demonstrated that α7 nAChR potentiation significantly inhibited the activation of microglia and astrocytes, as well as suppressed the release of proinflammatory cytokines in the spinal cord of rodent models of chronic pain. Nevertheless, how activation of α7 nAChR in neuronal and non-neuronal cells may reduce neuroinflammation in chronic pain remains elusive. Therefore, detailed mechanisms underlying the analgesic effects of α7 nAChR agonists and allosteric modulators must be clarified. For instance, activation of adenosine monophosphate-activated protein kinase (AMPK) signaling might be an interesting hypothesis accounting for the analgesic effects of α7 nAChR agonists and allosteric modulators. It has been frequently reported that potentiation of α7 nAChR elicits activation of AMPK signaling in various diseases (Hasan et al., 2018; Lin et al., 2019; Shao et al., 2019; Xu et al., 2021). Previous studies showed that AMPK activation significantly suppressed neuroinflammation in the central nervous system in chronic pain (Song et al., 2015; Zhang et al., 2021a; Wan et al., 2022). Recently, our laboratory demonstrated that induction of AMPK-mediated mitochondrial biogenesis plays a pivotal role in alleviating chronic pain (Sun et al., 2022). Therefore, it is worth finding whether activation of AMPK signaling contributes to the analgesic effects of α7 nAChR agonists and allosteric modulators.

Second, the conclusions of the studies mentioned above were based on male rodents only, which is a weakness of the work. Emerging evidence indicates a sex difference in susceptibility to chronic pain (Won et al., 2020). For instance, microglia are required for mechanical pain hypersensitivity in male mice, whereas female mice achieve similar levels of pain hypersensitivity using adaptive immune cells, likely T lymphocytes (Sorge et al., 2015). Recently, it was reported that α7 nAChR regulated hippocampal adult-neurogenesis in a sexually dimorphic fashion (Otto and Yakel, 2019). Therefore, future studies should determine the role of α7 nAChR in chronic pain in females.

Besides, although compelling evidence supports the pivotal role of α7 nAChR potentiation in chronic pain, what contributes to the decreased expression level of α7 nAChR in the pain transmission pathway remains unclear. More importantly, the mechanism behind agonist-induced upregulation of α7 nAChR is unknown. Therefore, the mechanisms underlying the decreased expression level of α7 nAChR in chronic pain and agonist-induced upregulation of α7 nAChR await further investigation.

Finally, despite the encouraging therapeutic efficacy of α7 nAChR agonists and allosteric modulators in chronic pain in preclinical studies, no relevant clinical trials are available. Notably, α7 nAChR agonists and allosteric modulators have shown good safety, tolerability, and efficacy profiles in other diseases such as schizophrenia (Zhang et al., 2012; Xia et al., 2020). However, concerns have been raised regarding the long-term administration of α7 nAChR agonists due to rapid desensitization of α7 nAChR, receptor selectivity issues, and the narrow window of antinociceptive effect *in vivo*. These limitations must be addressed before applying α7 nAChR as an analgesic in the clinic. Overall, these studies suggest that α7 nAChR is a promising therapeutic target for chronic pain, but more research regarding its detailed mechanisms is warranted.

## Author contributions

X-BT conceived the idea. Y-QZ and D-QL wrote and edited the manuscript. CL, A-JX, Y-KT, and WM reviewed and edited the manuscript. All authors read and approved the final manuscript.

## Funding

This work was supported by Grants from the National Natural Science Foundation of China (82001198, 82101310, and 81974170) and the Natural Science Foundation of Hubei Province (2021CFB341).

## Conflict of interest

The authors declare that the research was conducted in the absence of any commercial or financial relationships that could be construed as a potential conflict of interest.

## Publisher's note

All claims expressed in this article are solely those of the authors and do not necessarily represent those of their affiliated organizations, or those of the publisher, the editors and the reviewers. Any product that may be evaluated in this article, or claim that may be made by its manufacturer, is not guaranteed or endorsed by the publisher.

## References

[B1] Abbas M. Alzarea S. Papke R. L. Rahman S. (2019). The alpha7 nicotinic acetylcholine receptor positive allosteric modulator prevents lipopolysaccharide-induced allodynia, hyperalgesia, and TNF-alpha in the hippocampus in mice. Pharmacol. Rep. 71, 1168–1176. 10.1016/j.pharep.2019.07.001 31655281 PMC7745232

[B2] Abbas M. Alzarea S. Papke R. L. Rahman S. (2021). Effects of alpha7 nicotinic acetylcholine receptor positive allosteric modulator on BDNF, NKCC1, and KCC2 expression in the hippocampus following lipopolysaccharide-induced allodynia and hyperalgesia in a mouse model of inflammatory pain. CNS Neurol. Disord. Drug Targets 20, 366–377. 10.2174/1871527319666201230102616 33380307

[B3] Alsharari S. D. Freitas K. Damaj M. I. (2013). Functional role of alpha7 nicotinic receptor in chronic neuropathic and inflammatory pain: studies in transgenic mice. Biochem. Pharmacol. 86, 1201–1207. 10.1016/j.bcp.2013.06.018 23811428

[B4] Apryani E. Ali U. Wang Z. Y. Wu H. Y. Mao X. F. Ahmad K. A.. (2020). The spinal microglial IL-10/beta-endorphin pathway accounts for cinobufagin-induced mechanical antiallodynia in bone cancer pain following activation of alpha7-nicotinic acetylcholine receptors. J. Neuroinflamm. 17, 75. 10.1186/s12974-019-1616-z 32113469 PMC7049212

[B5] Arias H. R. Ghelardini C. Lucarini E. Tae H. S. Yousuf A. Marcovich I.. (2020). (E)-3-Furan-2-yl-N-p-tolyl-acrylamide and its derivative DM489 decrease neuropathic pain in mice predominantly by alpha7 nicotinic acetylcholine receptor potentiation. ACS Chem. Neurosci. 11, 3603–3614. 10.1021/acschemneuro.0c00476 33073974

[B6] Bagdas D. Gurun M. S. Flood P. Papke R. L. Damaj M. I. (2018a). New insights on neuronal nicotinic acetylcholine receptors as targets for pain and inflammation: a focus on alpha7 nAChRs. Curr. Neuropharmacol. 16, 415–425. 10.2174/1570159X15666170818102108 28820052 PMC6018191

[B7] Bagdas D. Meade J. A. Alkhlaif Y. Muldoon P. P. Carroll F. I. Damaj M. I. (2018b). Effect of nicotine and alpha-7 nicotinic modulators on visceral pain-induced conditioned place aversion in mice. Eur. J. Pain. 22, 1419–1427. 10.1002/ejp.1231 29633429 PMC6179949

[B8] Bagdas D. Sevdar G. Gul Z. Younis R. Cavun S. Tae H. S.. (2021). (E)-3-furan-2-yl-N-phenylacrylamide (PAM-4) decreases nociception and emotional manifestations of neuropathic pain in mice by alpha7 nicotinic acetylcholine receptor potentiation. Neurol. Res. 43, 1056–1068. 10.1080/01616412.2021.1949684 34281483

[B9] Bagdas D. Wilkerson J. L. Kulkarni A. Toma W. Alsharari S. Gul Z.. (2016). The alpha7 nicotinic receptor dual allosteric agonist and positive allosteric modulator GAT107 reverses nociception in mouse models of inflammatory and neuropathic pain. Br. J. Pharmacol. 173, 2506–2520. 10.1111/bph.13528 27243753 PMC4959951

[B10] Bagley E. E. Ingram S. L. (2020). Endogenous opioid peptides in the descending pain modulatory circuit. Neuropharmacology 173, 108131. 10.1016/j.neuropharm.2020.108131 32422213 PMC7313723

[B11] Beinat C. Banister S. D. Herrera M. Law V. Kassiou M. (2015). The therapeutic potential of alpha7 nicotinic acetylcholine receptor (alpha7 nAChR) agonists for the treatment of the cognitive deficits associated with schizophrenia. CNS Drugs 29, 529–542. 10.1007/s40263-015-0260-0 26242477

[B12] Brunori G. Schoch J. Mercatelli D. Ozawa A. Toll L. Cippitelli A. (2018). Influence of neuropathic pain on nicotinic acetylcholine receptor plasticity and behavioral responses to nicotine in rats. Pain 159, 2179–2191. 10.1097/j.pain.0000000000001318 29939964 PMC6193825

[B13] Burma N. E. Leduc-Pessah H. Fan C. Y. Trang T. (2017). Animal models of chronic pain: advances and challenges for clinical translation. J. Neurosci. Res. 95, 1242–1256. 10.1002/jnr.23768 27376591

[B14] Chen R. Yin C. Fang J. Liu B. (2021). The NLRP3 inflammasome: an emerging therapeutic target for chronic pain. J. Neuroinflamm. 18, 84. 10.1186/s12974-021-02131-0 33785039 PMC8008529

[B15] Chimienti F. Hogg R. C. Plantard L. Lehmann C. Brakch N. Fischer J.. (2003). Identification of SLURP-1 as an epidermal neuromodulator explains the clinical phenotype of Mal de Meleda. Hum. Mol. Genet. 12, 3017–3024. 10.1093/hmg/ddg320 14506129

[B16] Cordero-Erausquin M. Pons S. Faure P. Changeux J. P. (2004). Nicotine differentially activates inhibitory and excitatory neurons in the dorsal spinal cord. Pain 109, 308–318. 10.1016/j.pain.2004.01.034 15157692

[B17] Cornett E. M. Budish R. Latimer D. Hart B. Urman R. D. Kaye A. D. (2018). Management of Challenging Pharmacologic Issues in Chronic Pain and Substance Abuse Disorders. Anesthesiol. Clin. 36, 615–626. 10.1016/j.anclin.2018.07.009 30390782

[B18] Costa R. Motta E. M. Manjavachi M. N. Cola M. Calixto J. B. (2012). Activation of the alpha-7 nicotinic acetylcholine receptor (alpha7 nAchR) reverses referred mechanical hyperalgesia induced by colonic inflammation in mice. Neuropharmacology 63, 798–805. 10.1016/j.neuropharm.2012.06.004 22722030

[B19] Criado M. Balsera B. Mulet J. Sala S. Sala F. De La Torre-Martinez R.. (2016). 1,3-diphenylpropan-1-ones as allosteric modulators of alpha7 nACh receptors with analgesic and antioxidant properties. Future Med. Chem. 8, 731–749. 10.4155/fmc-2015-0001 27161515

[B20] Di Cesare Mannelli L. Pacini A. Matera C. Zanardelli M. Mello T. De Amici M.. (2014). Involvement of alpha7 nAChR subtype in rat oxaliplatin-induced neuropathy: effects of selective activation. Neuropharmacology 79, 37–48. 10.1016/j.neuropharm.2013.10.034 24225197

[B21] Di Cesare Mannelli L. Zanardelli M. Ghelardini C. (2013). Nicotine is a pain reliever in trauma- and chemotherapy-induced neuropathy models. Eur. J. Pharmacol. 711, 87–94. 10.1016/j.ejphar.2013.04.022 23648560

[B22] Dineley K. T. Pandya A. A. Yakel J. L. (2015). Nicotinic ACh receptors as therapeutic targets in CNS disorders. Trends Pharmacol. Sci. 36, 96–108. 10.1016/j.tips.2014.12.002 25639674 PMC4324614

[B23] Echeverria V. Yarkov A. Aliev G. (2016). Positive modulators of the alpha7 nicotinic receptor against neuroinflammation and cognitive impairment in Alzheimer's disease. Prog. Neurobiol. 144, 142–157. 10.1016/j.pneurobio.2016.01.002 26797042

[B24] Egea J. Buendia I. Parada E. Navarro E. Leon R. Lopez M. G. (2015). Anti-inflammatory role of microglial alpha7 nAChRs and its role in neuroprotection. Biochem. Pharmacol. 97, 463–472. 10.1016/j.bcp.2015.07.032 26232730

[B25] Freitas K. Carroll F. I. Damaj M. I. (2013a). The antinociceptive effects of nicotinic receptors alpha7-positive allosteric modulators in murine acute and tonic pain models. J. Pharmacol. Exp. Ther. 344, 264–275. 10.1124/jpet.112.197871 23115222 PMC3533419

[B26] Freitas K. Ghosh S. Ivy Carroll F. Lichtman A. H. Imad Damaj M. (2013b). Effects of alpha7 positive allosteric modulators in murine inflammatory and chronic neuropathic pain models. Neuropharmacology 65, 156–164. 10.1016/j.neuropharm.2012.08.022 23079470 PMC3521074

[B27] Freitas K. Negus S. S. Carroll F. I. Damaj M. I. (2013c). *In vivo* pharmacological interactions between a type II positive allosteric modulator of alpha7 nicotinic ACh receptors and nicotinic agonists in a murine tonic pain model. Br. J. Pharmacol. 169, 567–579. 10.1111/j.1476-5381.2012.02226.x 23004024 PMC3682705

[B28] Goldstick J. E. Guy G. P. Losby J. L. Baldwin G. T. Myers M. G. Bohnert A. S. B. (2022). Patterns in nonopioid pain medication prescribing after the release of the 2016 guideline for prescribing opioids for chronic pain. JAMA Netw. Open 5, e2216475. 10.1001/jamanetworkopen.2022.16475 35687334 PMC9187961

[B29] Gong S. Liang Q. Zhu Q. Ding D. Yin Q. Tao J.. (2015). Nicotinic acetylcholine receptor alpha7 subunit is involved in the cobratoxin-induced antinociception in an animal model of neuropathic pain. Toxicon 93, 31–36. 10.1016/j.toxicon.2014.11.222 25447771

[B30] Han Q. Q. Yin M. Wang Z. Y. Liu H. Ao J. P. Wang Y. X. (2020). Cynandione A alleviates neuropathic pain through alpha7-nAChR-dependent IL-10/beta-endorphin signaling complexes. Front. Pharmacol. 11, 614450. 10.3389/fphar.2020.614450 33584292 PMC7873367

[B31] Hasan M. K. Friedman T. C. Sims C. Lee D. L. Espinoza-Derout J. Ume A.. (2018). alpha7-nicotinic acetylcholine receptor agonist ameliorates nicotine plus high-fat diet-induced hepatic steatosis in male mice by inhibiting oxidative stress and stimulating AMPK signaling. Endocrinology 159, 931–944. 10.1210/en.2017-00594 29272360 PMC5776480

[B32] Hone A. J. Mcintosh J. M. (2018). Nicotinic acetylcholine receptors in neuropathic and inflammatory pain. FEBS Lett. 592, 1045–1062. 10.1002/1873-3468.12884 29030971 PMC5899685

[B33] Ji L. Chen Y. Wei H. Feng H. Chang R. Yu D.. (2019). Activation of alpha7 acetylcholine receptors reduces neuropathic pain by decreasing dynorphin A release from microglia. Brain Res. 1715, 57–65. 10.1016/j.brainres.2019.03.016 30898676

[B34] Ji R. R. Nackley A. Huh Y. Terrando N. Maixner W. (2018). Neuroinflammation and central sensitization in chronic and widespread pain. Anesthesiology 129, 343–366. 10.1097/ALN.0000000000002130 29462012 PMC6051899

[B35] Ji R. R. Xu Z. Z. Gao Y. J. (2014). Emerging targets in neuroinflammation-driven chronic pain. Nat. Rev. Drug Discov. 13, 533–548. 10.1038/nrd4334 24948120 PMC4228377

[B36] Jiang B. C. Liu T. Gao Y. J. (2020). Chemokines in chronic pain: cellular and molecular mechanisms and therapeutic potential. Pharmacol. Ther. 212, 107581. 10.1016/j.pharmthera.2020.107581 32450191

[B37] Kelso M. L. Oestreich J. H. (2012). Traumatic brain injury: central and peripheral role of alpha7 nicotinic acetylcholine receptors. Curr. Drug Targets 13, 631–636. 10.2174/138945012800398964 22300031

[B38] Khasabov S. G. Rogness V. M. Beeson M. B. Vulchanova L. Yuan L. L. Simone D. A.. (2021). The nAChR chaperone TMEM35a (NACHO) contributes to the development of hyperalgesia in mice. Neuroscience 457, 74–87. 10.1016/j.neuroscience.2020.12.027 33422618 PMC7897319

[B39] Lin Z. H. Li Y. C. Wu S. J. Zheng C. Lin Y. Z. Lian H.. (2019). Eliciting alpha7-nAChR exerts cardioprotective effects on ischemic cardiomyopathy *via* activation of AMPK signalling. J. Cell Mol. Med. 23, 4746–4758. 10.1111/jcmm.14363 31062470 PMC6584557

[B40] Liu D. Q. Zhou Y. Q. Gao F. (2019). Targeting cytokines for morphine tolerance: a narrative review. Curr. Neuropharmacol. 17, 366–376. 10.2174/1570159X15666171128144441 29189168 PMC6482476

[B41] Liu Y. Xu S. Zhang H. Qian K. Huang J. Gu X.. (2021). Stimulation of alpha7-nAChRs coordinates autophagy and apoptosis signaling in experimental knee osteoarthritis. Cell Death Dis. 12, 448. 10.1038/s41419-021-03726-4 33953172 PMC8100296

[B42] Loram L. C. Harrison J. A. Chao L. Taylor F. R. Reddy A. Travis C. L.. (2010). Intrathecal injection of an alpha seven nicotinic acetylcholine receptor agonist attenuates gp120-induced mechanical allodynia and spinal proinflammatory cytokine profiles in rats. Brain Behav. Immun. 24, 959–967. 10.1016/j.bbi.2010.03.008 20353818 PMC2902784

[B43] Loram L. C. Taylor F. R. Strand K. A. Maier S. F. Speake J. D. Jordan K. G.. (2012). Systemic administration of an alpha-7 nicotinic acetylcholine agonist reverses neuropathic pain in male Sprague Dawley rats. J. Pain 13, 1162–1171. 10.1016/j.jpain.2012.08.009 23182225 PMC5654381

[B44] Ma K. G. Qian Y. H. (2019). Alpha 7 nicotinic acetylcholine receptor and its effects on Alzheimer's disease. Neuropeptides 73, 96–106. 10.1016/j.npep.2018.12.003 30579679

[B45] Matta J. A. Gu S. Davini W. B. Bredt D. S. (2021). Nicotinic acetylcholine receptor redux: discovery of accessories opens therapeutic vistas. Science 373, abg6539. 10.1126/science.abg6539 34385370

[B46] Matthias M. S. Talib T. L. Huffman M. A. (2020). Managing chronic pain in an opioid crisis: what is the role of shared decision-making? Health Commun. 35, 1239–1247. 10.1080/10410236.2019.1625000 31179769 PMC6901808

[B47] Medhurst S. J. Hatcher J. P. Hille C. J. Bingham S. Clayton N. M. Billinton A.. (2008). Activation of the alpha7-nicotinic acetylcholine receptor reverses complete freund adjuvant-induced mechanical hyperalgesia in the rat *via* a central site of action. J. Pain 9, 580–587. 10.1016/j.jpain.2008.01.336 18420461

[B48] Micheli L. Rajagopalan R. Lucarini E. Toti A. Parisio C. Carrino D.. (2021). Pain relieving and neuroprotective effects of non-opioid compound, DDD-028, in the rat model of paclitaxel-induced neuropathy. Neurotherapeutics 18, 2008–2020. 10.1007/s13311-021-01069-8 34312766 PMC8608957

[B49] Mizrachi T. Vaknin-Dembinsky A. Brenner T. Treinin M. (2021). Neuroinflammation modulation *via* alpha7 nicotinic acetylcholine receptor and its chaperone, RIC-3. Molecules 26, 139. 10.3390/molecules26206139 34684720 PMC8539643

[B50] Moriwaki Y. Watanabe Y. Shinagawa T. Kai M. Miyazawa M. Okuda T.. (2009). Primary sensory neuronal expression of SLURP-1, an endogenous nicotinic acetylcholine receptor ligand. Neurosci. Res. 64, 403–412. 10.1016/j.neures.2009.04.014 19409425

[B51] Otto S. L. Yakel J. L. (2019). The alpha7 nicotinic acetylcholine receptors regulate hippocampal adult-neurogenesis in a sexually dimorphic fashion. Brain Struct. Funct. 224, 829–846. 10.1007/s00429-018-1799-6 30515567 PMC6432768

[B52] Papke R. L. Bagdas D. Kulkarni A. R. Gould T. Alsharari S. D. Thakur G. A.. (2015). The analgesic-like properties of the alpha7 nAChR silent agonist NS6740 is associated with non-conducting conformations of the receptor. Neuropharmacology 91, 34–42. 10.1016/j.neuropharm.2014.12.002 25497451 PMC4312719

[B53] Papke R. L. Chojnacka K. Horenstein N. A. (2014). The minimal pharmacophore for silent agonism of the alpha7 nicotinic acetylcholine receptor. J. Pharmacol. Exp. Ther. 350, 665–680. 10.1124/jpet.114.215236 24990939 PMC4152879

[B54] Papke R. L. Horenstein N. A. (2021). Therapeutic targeting of alpha7 nicotinic acetylcholine receptors. Pharmacol. Rev. 73, 1118–1149. 10.1124/pharmrev.120.000097 34301823 PMC8318519

[B55] Papke R. L. Lindstrom J. M. (2020). Nicotinic acetylcholine receptors: conventional and unconventional ligands and signaling. Neuropharmacology 168, 108021. 10.1016/j.neuropharm.2020.108021 32146229 PMC7610230

[B56] Quadri M. Bagdas D. Toma W. Stokes C. Horenstein N. A. Damaj M. I.. (2018). The antinociceptive and anti-inflammatory properties of the alpha7 nAChR weak partial agonist p-CF3 N, N-diethyl-N'-phenylpiperazine. J. Pharmacol. Exp. Ther. 367, 203–214. 10.1124/jpet.118.249904 30111636 PMC7593094

[B57] Quik M. Zhang D. Mcgregor M. Bordia T. (2015). Alpha7 nicotinic receptors as therapeutic targets for Parkinson's disease. Biochem. Pharmacol. 97, 399–407. 10.1016/j.bcp.2015.06.014 26093062 PMC4600450

[B58] Richardson E. J. Ness T. J. Redden D. T. Stewart C. C. Richards J. S. (2012). Effects of nicotine on spinal cord injury pain vary among subtypes of pain and smoking status: results from a randomized, controlled experiment. J. Pain 13, 1206–1214. 10.1016/j.jpain.2012.09.005 23107508

[B59] Shao B. Z. Wang S. L. Fang J. Li Z. S. Bai Y. Wu K. (2019). Alpha7 nicotinic acetylcholine receptor alleviates inflammatory bowel disease through induction of AMPK-mTOR-p70S6K-mediated autophagy. Inflammation 42, 1666–1679. 10.1007/s10753-019-01027-9 31236857

[B60] Song H. Han Y. Pan C. Deng X. Dai W. Hu L.. (2015). Activation of adenosine monophosphate-activated protein kinase suppresses neuroinflammation and ameliorates bone cancer pain: involvement of inhibition on mitogen-activated protein kinase. Anesthesiology 123, 1170–1185. 10.1097/ALN.0000000000000856 26378398

[B61] Sorge R. E. Mapplebeck J. C. Rosen S. Beggs S. Taves S. Alexander J. K.. (2015). Different immune cells mediate mechanical pain hypersensitivity in male and female mice. Nat. Neurosci. 18, 1081–1083. 10.1038/nn.4053 26120961 PMC4772157

[B62] Sun J. Song F. H. Wu J. Y. Zhang L. Q. Li D. Y. Gao S. J.. (2022). Sestrin2 overexpression attenuates osteoarthritis pain *via* induction of AMPK/PGC-1alpha-mediated mitochondrial biogenesis and suppression of neuroinflammation. Brain Behav. Immun. 102, 53–70. 10.1016/j.bbi.2022.02.015 35151829

[B63] Sun R. Liu Y. Hou B. Lei Y. Bo J. Zhang W.. (2019). Perioperative activation of spinal alpha7 nAChR promotes recovery from preoperative stress-induced prolongation of postsurgical pain. Brain Behav. Immun. 79, 294–308. 10.1016/j.bbi.2019.02.017 30797046

[B64] Sun R. Zhang W. Bo J. Zhang Z. Lei Y. Huo W.. (2017). Spinal activation of alpha7-nicotinic acetylcholine receptor attenuates posttraumatic stress disorder-related chronic pain *via* suppression of glial activation. Neuroscience 344, 243–254. 10.1016/j.neuroscience.2016.12.029 28039041

[B65] Teng P. Liu Y. Dai Y. Zhang H. Liu W. T. Hu J. (2019). Nicotine attenuates osteoarthritis pain and matrix metalloproteinase-9 expression *via* the alpha7 nicotinic acetylcholine receptor. J. Immunol. 203, 485–492. 10.4049/jimmunol.1801513 31152077

[B66] Toma W. Kyte S. L. Bagdas D. Jackson A. Meade J. A. Rahman F.. (2019). The alpha7 nicotinic receptor silent agonist R-47 prevents and reverses paclitaxel-induced peripheral neuropathy in mice without tolerance or altering nicotine reward and withdrawal. Exp. Neurol. 320, 113010. 10.1016/j.expneurol.2019.113010 31299179 PMC6708482

[B67] Toma W. Ulker E. Alqasem M. Alsharari S. D. Mcintosh J. M. Damaj M. I. (2020). Behavioral and molecular basis of cholinergic modulation of pain: focus on nicotinic acetylcholine receptors. Curr. Top. Behav. Neurosci. 45, 153–166. 10.1007/7854_2020_135 32468494

[B68] Umana I. C. Daniele C. A. Miller B. A. Abburi C. Gallagher K. Brown M. A.. (2017). Nicotinic modulation of descending pain control circuitry. Pain 158, 1938–1950. 10.1097/j.pain.0000000000000993 28817416 PMC5873975

[B69] Wan L. Jia R. M. Ji L. L. Qin X. M. Hu L. Hu F.. (2022). AMPK-autophagy-mediated inhibition of microRNA-30a-5p alleviates morphine tolerance *via* SOCS3-dependent neuroinflammation suppression. J. Neuroinflamm. 19, 25. 10.1186/s12974-022-02384-3 35093117 PMC8800317

[B70] Wang D. Yang H. Liang Y. Wang X. Du X. Li R.. (2019). Antinociceptive effect of spirocyclopiperazinium salt compound DXL-A-24 and the underlying mechanism. Neurochem. Res. 44, 2786–2795. 10.1007/s11064-019-02899-x 31691883

[B71] Won S. Park K. Lim H. Lee S. J. (2020). Sexual dimorphism in cognitive disorders in a murine model of neuropathic pain. Behav. Brain Funct. 16, 1. 10.1186/s12993-019-0164-0 31901234 PMC6942364

[B72] Xia L. Liu L. Hong X. Wang D. Wei G. Wang J.. (2020). One-day tropisetron treatment improves cognitive deficits and P50 inhibition deficits in schizophrenia. Neuropsychopharmacology 45, 1362–1368. 10.1038/s41386-020-0685-0 32349117 PMC7297960

[B73] Xu Z. Q. Zhang J. J. Kong N. Zhang G. Y. Ke P. Han T.. (2021). Autophagy is involved in neuroprotective effect of Alpha7 nicotinic acetylcholine receptor on ischemic stroke. Front. Pharmacol. 12, 676589. 10.3389/fphar.2021.676589 33995108 PMC8117007

[B74] Yang H. Sun Q. Liang Y. Jiang Y. Li R. Ye J. (2018). Antinociception of the spirocyclopiperazinium salt compound LXM-15 *via* activating alpha7 nAChR and M4 mAChR and inhibiting CaMKIIalpha/cAMP/CREB/CGRP signalling pathway in mice. Regul. Toxicol. Pharmacol. 94, 108–114. 10.1016/j.yrtph.2018.01.012 29353067

[B75] Yang T. Zhou Y. Zhang W. Zhang L. Chen S. Chen C.. (2021). The spinal alpha7-nicotinic acetylcholine receptor contributes to the maintenance of cancer-induced bone pain. J. Pain Res. 14, 441–452. 10.2147/JPR.S286321 33623426 PMC7894822

[B76] Zhang L. Q. Zhang W. Li T. Yang T. Yuan X. Zhou Y.. (2021a). GLP-1R activation ameliorated novel-object recognition memory dysfunction *via* regulating hippocampal AMPK/NF-kappaB pathway in neuropathic pain mice. Neurobiol. Learn Mem. 182, 107463. 10.1016/j.nlm.2021.107463 34015440

[B77] Zhang X. Xu F. Wang L. Li J. Zhang J. Huang L. (2021b). The role of dorsal root ganglia alpha-7 nicotinic acetylcholine receptor in complete Freund's adjuvant-induced chronic inflammatory pain. Inflammopharmacology 29, 1487–1501. 10.1007/s10787-021-00873-0 34514543

[B78] Zhang X. Y. Liu L. Liu S. Hong X. Chen D. C. Xiu M. H.. (2012). Short-term tropisetron treatment and cognitive and P50 auditory gating deficits in schizophrenia. Am. J. Psychiatry 169, 974–981. 10.1176/appi.ajp.2012.11081289 22952075

[B79] Zhang Y. Sevilla A. Weller R. Wang S. Gitlin M. C. Candiotti K. A. (2021c). The role of alpha7-nicotinic acetylcholine receptor in a rat model of chronic nicotine-induced mechanical hypersensitivity. Neurosci. Lett. 743, 135566. 10.1016/j.neulet.2020.135566 33352289

[B80] Zhou Y. Q. Liu D. Q. Chen S. P. Sun J. Zhou X. R. Xing C.. (2019). The role of CXCR3 in neurological diseases. Curr. Neuropharmacol. 17, 142–150. 10.2174/1570159X15666171109161140 29119926 PMC6343204

[B81] Zhu S. Huang S. Xia G. Wu J. Shen Y. Wang Y.. (2021). Anti-inflammatory effects of alpha7-nicotinic ACh receptors are exerted through interactions with adenylyl cyclase-6. Br. J. Pharmacol. 178, 2324–2338. 10.1111/bph.15412 33598912

